# Zinc Transporter-3 Knockout Mice Demonstrate Age-Dependent Alterations in the Metalloproteome

**DOI:** 10.3390/ijms21030839

**Published:** 2020-01-28

**Authors:** Sara M. Hancock, Stuart D. Portbury, Adam P. Gunn, Blaine R. Roberts, Ashley I. Bush, Paul A. Adlard

**Affiliations:** 1 The Florey Institute of Neuroscience and Mental Health, The University of Melbourne, Melbourne, VIC 3010, Australia; sara.m.tavasz@gmail.com (S.M.H.); stuart.portbury@florey.edu.au (S.D.P.); adam.gunn@canberra.edu.au (A.P.G.); blaine.roberts@emory.edu (B.R.R.); ashley.bush@florey.edu.au (A.I.B.); 2 The Melbourne Dementia Research Centre, Parkville, VIC 3052, Australia

**Keywords:** Zinc, ZnT3, Alzheimer’s disease, metals, ageing

## Abstract

Metals are critical cellular elements that are involved in a variety of cellular processes, with recent literature demonstrating that zinc, and the synaptic zinc transporter (ZnT3), are specifically involved in learning and memory and may also be key players in age-related neurodegenerative disorders such as Alzheimer’s disease. Whilst the cellular content and location of metals is critical, recent data has demonstrated that the metalation state of proteins is a determinant of protein function and potential toxicity. As we have previously reported that ZnT3 knockout (KO) mice have deficits in total zinc levels at both 3 and 6 months of age, we were interested in whether there might be changes in the metalloproteomic profile in these animals. To do this, we utilised size exclusion chromatography-inductively coupled plasma mass spectrometry (SEC-ICP-MS) and examined hippocampal homogenates from ZnT3 KO and age-matched wild-type mice at 3, 6 and 18 months of age. Our data suggest that there are alterations in specific metal binding proteins, for zinc, copper and iron all being modulated in the ZnT3 KO mice compared to wild-type (WT). These data suggest that ZnT3 KO mice may have impairments in the levels or localisation of multiple transition metals, and that copper- and iron-dependent cellular pathways may also be impacted in these mice.

## 1. Introduction

One of the key cellular processes that becomes dysregulated with age and participates both directly and indirectly in age-related dysfunction, is metal homeostasis [1,2,3]. Zinc is one such metal that is critical for the activity of a variety of enzymes, transcription factors and cellular processes, the levels of which are tightly controlled by a family of zinc import/export proteins that have a distinct pattern of cellular and tissue localization as well as expression [4,5]. These principally comprise the zinc transporter (ZnT) and zinc-regulated and iron-regulated transporter proteins (ZIPs), as well as other zinc “handling” proteins such as metallothioneins. Approximately 10%–15% of brain zinc exists as chelatable zinc primarily within synaptic vesicles at glutamatergic synapses, highlighting its importance in synaptic plasticity/cognition. Our discoveries highlighted the critical importance of this pool of zinc, and the synaptic zinc transporter (ZnT3 (SLC30A3)), in cognition [6,7,8,9]. Specifically, we reported that there were age-dependent deficits in learning and memory in the ZnT3 knockout (KO) mice that were manifest at 6 months of age (but not at 3 months), and which were associated with significant deficits in a suite of key hippocampal proteins involved in learning and memory. The ZnT3 KO mice also had significantly decreased hippocampal zinc levels at both 3 (−39%) and 6 (−32%) months of age compared to age-matched wild-type (WT) mice, which whilst of greater magnitude than initially reported for the whole brain, is consistent with our focus on the hippocampus and the loss of the synaptic vesicular zinc pool [10,11,12,13]. 

Whilst such a bulk change in metals may be important, it is also known that the complement of metals associated with a given protein is important and can affect protein function, as in the case of Cu/Zn-superoxide dismutase (SOD1) [14]. In order to gain further insight into the metalloproteome in the ZnT3 KO mice, we utilised size-exclusion inductively coupled plasma mass spectrometry (SEC-ICP-MS) to examine hippocampal homogenates from ZnT3 KO and age-matched WT mice at 3, 6 and 18 months of age. This SEC-ICP-MS technique allows for the chromatographic separation of proteins from complex mixtures using a size exclusion column (SEC) and the subsequent assessment of the elemental content of those fractions using inductively coupled plasma mass spectrometry (ICPMS). This gives rise to an elution profile (the chromatogram) where the peaks represent the metal content of protein/s of a given molecular weight.

## 2. Materials and Methods

### 2.1. Animal Ethics

All animal experimentation procedures were approved by the Florey Institute of Neuroscience Animal Ethics Committee and conducted in accordance with the Prevention of Cruelty to Animals Act and the NH&MRC Code of Practice for the Use of Animals for Scientific Purposes.

### 2.2. Animals

Animals were maintained in standard group-housed laboratory conditions and were provided regular rodent chow and water ad libitum (the metal content of the rodent chow is reported as: 51 mg/kg iron, 60 mg/kg zinc and 10 mg/kg copper; the tap water has not been analysed). Animals were culled at three timepoints; 3, 6 and 18 months of age. The three-month-old cohort consisted of 12 knockout (male *n* = 6, female *n* = 6) and 12 wild-type (male *n* = 6, female *n* = 6) mice. The six-month-old cohort consisted of 12 knockout (male *n* = 6, female *n* = 6) and 12 wild-type (male *n* = 6, female n = 6) mice. The eighteen-month-old cohort consisted of 14 knockout (male *n* = 6, female *n* = 8) and 10 wild-type (male *n* = 7, female *n* = 3) mice. Mice were anaesthetized with Buprenorphine (80 mg/kg) via intraperitoneal injection before a 50 mL transcardial perfusion with ice cold PBS, followed by decapitation. Whole brain was removed from the skull and hemisected before microdissection of the hippocampus. Tissue was stored at −80 °C until further processing.

### 2.3. SEC-ICP-MS

SEC-ICP-MS analysis was performed using the previously described method [15]. Whole brain tissue samples were homogenized by probe sonication (3 rounds of sonication for 15 seconds on ice, 40% amplitude) in 1 mL of homogenization buffer (Dulbecco’s PBS with EDTA free proteinase inhibitor cocktails 2 and 3; 1:500; Roche) and centrifuged at 100,000× *g* for 30 minutes at 4 °C. The supernatant was collected, and both the pellet and supernatant were stored at −80 °C until further use. Samples of 100 µg of soluble protein were chromatographically separated using a BioSEC-3 column 3 μm, 4.6 × 300 mm (pore size 150 Å, MW range 0.5–150 kDa) (Agilent, VIC, Australia) with 200 mM ammonium nitrate containing internal standard (^133^Cs, ^121^Sb; 10 μg L^−1^ each) at a flow rate of 0.4 mL/min. The HPLC was directly connected to a MicroMist nebulizer (Glass Expansion, Australia) fitted to an Agilent technologies 7700 ICP-MS. Helium was used as the collision gas with all elements. The following elements were analysed: ^56^Fe, ^63^Cu and ^66^Zn. Baseline correction of the traces was conducted by subtracting the average of the first 50 data points which represents the background contribution of metal from the LC system and buffer combined. The peaks of the SEC chromatographs were integrated base on time as the peaks were not baseline resolved. The start and stop of the integration is marked on the 3 month time point graph in each of the figures. 

### 2.4. Statistical Analysis

Statistical analysis was carried out using Prism 8 (Graph-Pad) software. Before application of a one-way ANOVA with Sidak’s multiple comparisons correction, we tested if the data were consistent with a sampling from a normal Gaussian distribution using the Shapiro-Wilk test [16]. The limited group sizes required that the data were not separated by gender. However, as there have been differences reported in behavioural phenotypes across males and females in the ZnT3 KO mice [17], future studies should be sufficiently powered to assess whether gender also presents a confound at the level of the metalloproteome.

## 3. Results

### SEC-ICP-MS

**Figure 1:**
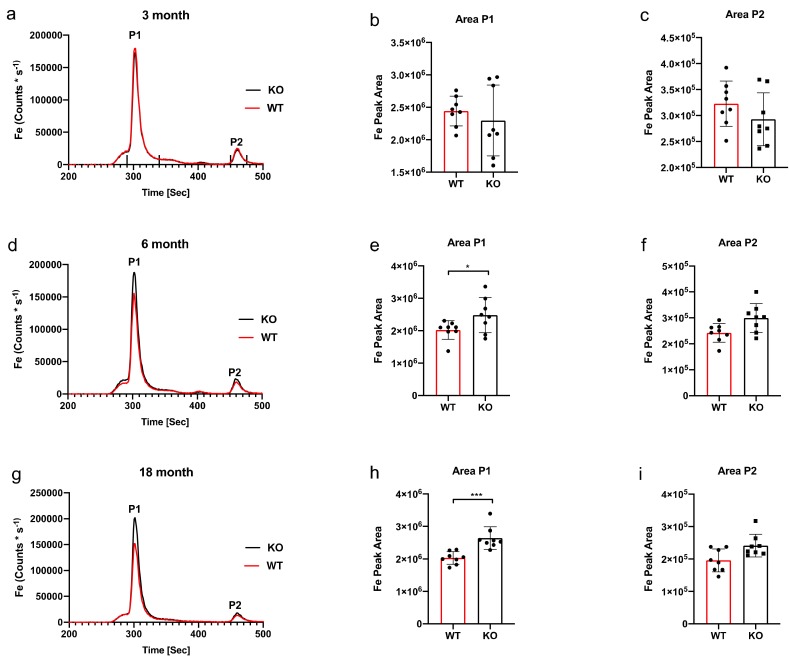
Inductively coupled plasma mass spectrometry (ICPMS) and area under the curve Fe analysis. No significant difference in Fe was observed between wild-type (WT) and knockout (KO) mice at the 3-month time point (**a**) further reflected in the area under the curve (AUC) analysis for peak 1 (**b**) and peak 2 (**c**). The ticks above the x-axis indicate the integration windows for the AUC analysis. There was a decrease in Fe observed in the ICPMS chromatogram (**d**) at the 6-month time point that was revealed to be significant for peak 1 (* *p* = 0.01) (**e**) and no change for peak 2 (**f**). At the 18-month time point there was a decrease in Fe observed in the ICPMS chromatogram (**g**) that was revealed to be significant for peak 1 (*** *p* = < 0.0001) (**h**) and no change for peak 2 (**i**). Dot plots and bar graph show the mean and SD, peak area statistical comparison one-way ANOVA Sidak’s multiple comparison test.

Iron (Fe) levels, from ZnT3 knockout and wild type litter mate brain samples, were assessed via liquid chromatography-inductively coupled-plasma mass spectrometry (SEC-ICP-MS) at 3-, 6- and 18- months of age. Fe56 was eluted as a single major peak at all time points measured. Based on the elution profile of iron-protein complexes previously published [15] and also the ferritin standard that was run, we conclude that peak 1 is most likely associated with ferritin (ferritin-iron). Due to the complexity of the tissue analysed, however, more than one protein may be associated with the peak observed in the chromatogram. At the three-month time point no difference was observed on the chromatogram (Figure 1a), an observation that was further verified by an area under the curve (AUC) analysis for peak 1 (Figure 1b) and peak 2 (Figure 1c). However, the 6-month time point chromatogram (Figure 1d) showed an elevation of Fe56 at peak 1 for the knockout mice. Peak 1 was shown to be significant by AUC analysis (Figure 1e), representing a 17% increase of Fe56 in the knockout mice, but peak 2 was not significantly changed (Figure 1f). An elevation of Fe56 in knockout mice was also observed in the 18-month chromatogram (Figure 1g). Peak 1 AUC analysis revealed a significant 22% increase (Figure 1h) in Fe56 for knockout mice.

**Figure 2:**
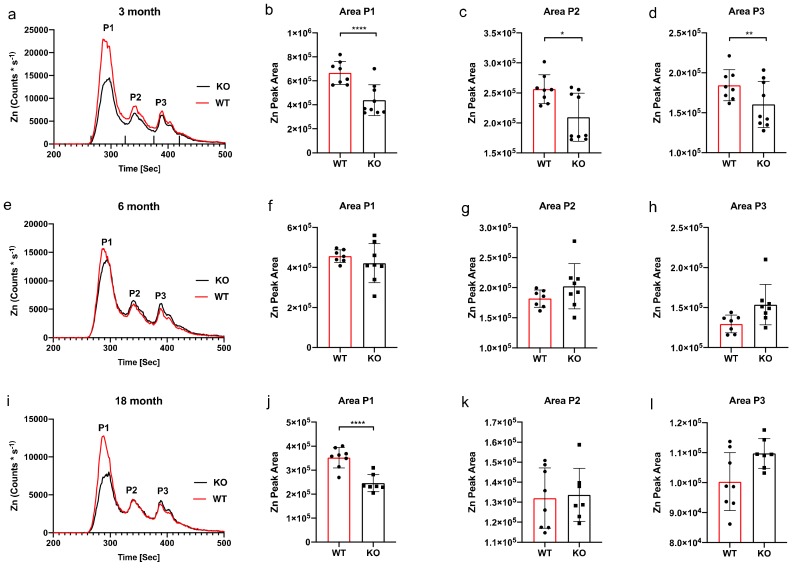
ICPMS and area under the curve Zn66 analysis. A difference in Zn was observed between WT and KO mice at the 3-month time point chromatogram (**a**). AUC analysis of peak 1 (**b**) revealed a significant decrease (**** *p* < 0.0001) between WT and KO, as did peak 2 (** *p* = 0.04) (**c**) and no significant difference for peak 3 (**d**). The ticks above the x-axis indicate the integration windows for the AUC analysis. No significant difference in Zn was observed between WT and KO mice at the 6-month time point (**e**), a result reflected in the analysis of the peak area for each major peak; peak 1 (**f**), peak 2 (**g**) and peak 3 (**h**). There was a decrease observed in the ICPMS chromatogram peak 1 for KO mice at the 18-month time point (**i**) that was revealed to be significant (* *p* < 0.0001) (**j**). Peak 2 (**k**) and peak 3 (**l**) revealed no significant differences between WT and KO. Dot plots and bar graph show the mean and SD, peak area statistical comparison one-way ANOVA Sidak’s multiple comparison test.

SEC-ICP-MS eluted three specific Zn66 peaks at all time points measured. At the three-month time-period the chromatogram revealed an obvious reduction of Zn66 in the knockout group at peaks 1, 2 and 3 (Figure 2a). AUC analysis of peak 1 revealed a significant 56% reduction in Zn66 (Figure 2b), a significant 28% reduction peak 2 (Figure 2c) and a significant 20% reduction in peak 3 (Figure 2d). However, the six-month age group chromatogram did not indicate obvious changes between wild type and knockout (Figure 2e), and indeed, this was reflected in the AUC analysis where peak 1 (Figure 2f), peak 2 (Figure 2g) and peak 3 (Figure 2h) were shown to have no significant differences. The 18-month chromatogram revealed a significant 43% reduction of Zn66 at peak 1 (Figure 2i), which was proven to be significant upon AUC analysis (Figure 2j); however AUC analysis of peak 2 (Figure 2k) and peak 3 (Figure 2l) were shown to have no significant differences.

**Figure 3:**
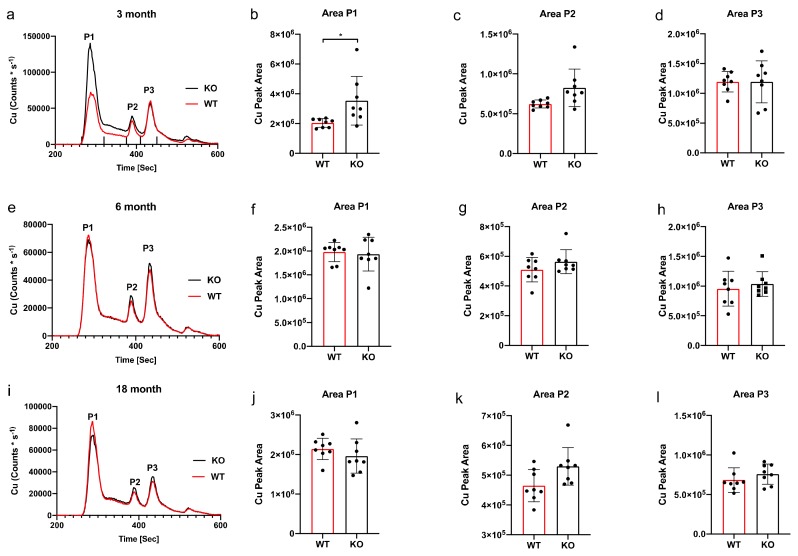
ICPMS and area under the curve Cu63 analysis. A difference in Cu was observed between WT and KO mice at the 3-month time point chromatogram (**a**). The ticks above the x-axis indicate the integration windows for the AUC analysis. Analysis of peak area 1 (**b**) revealed a significant decrease (* *p* = 0.02) between WT and KO, however peaks 2 and 3 revealed no difference (**c**,**d**). No significant difference in Cu was observed between WT and KO mice at the 6-month time point chromatogram (**e**). A result reflected in the area under the curve (AUC) analysis of peak 1 (**f**), peak 2 (**g**) and peak 3 (**h**). No significant difference in Zn was observed between WT and KO mice at the 6-month time point chromatogram (**i**). A result reflected in the area under the curve (AUC) analysis of peak 1 (**j**), peak 2 (**k**) and peak 3 (**l**). Dot plots and bar graph show the mean and SD, peak area statistical comparison one-way ANOVA Sidak’s multiple comparison test.

SEC-ICP-MS showed Cu63 eluted as three distinct major peaks at each time point measured. Whilst peaks 1 and 2 require further characterisation, peak 3 may be associated with superoxide dismutase (SOD), which plays an important role in protecting against oxidative damage, based on previously published data [15]. For the three-month time point, the chromatogram revealed an increase in Cu63 at peak 1 only (Figure 3a). AUC analysis of peak 1 confirmed a significant 56% increase in Cu63 (Figure 3b); peaks 2 and 3 were unchanged (Figure 3c,d). The 6-month chromatogram (Figure 3e) showed no obvious difference between wild type and knockout mice at any peak elution, which was further verified by AUC analysis demonstrating no significant changes at peak 1 (Figure 3f), peak 2 (Figure 3g) and peak 3 (Figure 3h). The 18-month chromatogram (Figure 3i) also showed no obvious difference between wild type and knockout mice at any peak elution, and this was further verified by AUC analysis demonstrating no significant changes at peak 1 (Figure 3j), peak 2 (Figure 3k) and peak 3 (Figure 3l).

## 4. Discussion

It has long been known that the homeostasis of metal ions is a critical requirement for normal cellular health. In the last few decades the role of metals in pathological ageing has also gained increasing attention, with strong preclinical evidence for their role in the onset and progression of numerous neurodegenerative diseases such as Huntington’s disease, schizophrenia, Parkinson’s disease and Alzheimer’s disease [18]. More recently, utilising the ZnT3 KO mouse line and other methods, we and others have also demonstrated a key role for zinc in “normal” synaptic plasticity and learning/memory via interactions on key neuronal signalling cascades and proteins [7,8,9,19,20,21,22]. Ultimately, the role of zinc in higher order cognitive function may well intersect with its effect on these and other age- and disease-related pathways. In order to further characterize the ZnT3 KO mouse line, which lacks zinc at the glutamatergic synapse, we have utilised SEC-ICP-MS to undertake an examination of the age-dependent changes in the metalloproteome in these mice. We demonstrate for the first time here that there are distinct changes not only in zinc, but also in the iron and to a lesser extent the copper, metalloproteome across age in the ZnT3 KO animals.

Changes to the normal metal content of a cell or tissue can occur through a variety of mechanisms that may be related to dietary, genetic or other pathways, which can also include metal:metal interactions that affect levels of a given element. Such direct and indirect changes in metals can all precipitate or potentiate biological outcomes. Whilst the cellular levels and distribution of metal is an important factor, recent evidence has also highlighted the notion that the amount of metal associated with a protein, i.e., the metalation state, may be an important determinant of protein function/toxicity. Two examples include metallothionein (MT) and superoxide dismutase. Metallothioneins are a family of proteins that have a capacity to bind various metals, including copper, zinc and cadmium. This sequestration of metals gives MTs a role in metal ion homeostasis and protection from the toxic effects of metals such as arsenic and lead. They also help regulate oxidative stress, and have reported roles in various diseases. One such disease is AD, where it has been reported that there is a “metal-swap” event with zinc-bound MT-3 (Zn_7_MT-3) such that MT-3 removes copper from aggregated and soluble β-amyloid-copper complexes (in turn exchanging metal species within MT-3, going from Zn_7_MT-3 to Cu_4_Zn_4_MT-3; β-amyloid is considered a primary toxic moiety in AD, and is the primary constituent of the β-amyloid plaque that characterizes the AD brain) to then prevent ROS production and subsequent cellular toxicity [23]. These effects are not seen with fully copper-laden MT-3. Thus, the metal content of MT-3 is a critical determinant of its ability to interact in this AD-related pathway that may be involved in disease pathogenesis. It is a similar scenario for the antioxidant enzyme copper-zinc superoxide dismutase (Cu, Zn-SOD), which exists as two isoforms (SOD1 and SOD3, having activity primarily in the cytoplasm and extracellular space respectively). SOD1 is implicated in the pathogenesis of amyotrophic lateral sclerosis [24], a rare disease that primarily results in the degeneration of upper and lower motor neurons leading to muscle paralysis and atrophy, but which is also associated with other non-motor symptoms [25,26]. SOD1 mutations account for ~15%–30% of familial ALS cases, depending upon the specific population. Whilst there is good evidence that the level of mutant SOD1 correlates well with disease in preclinical models, recent evidence suggests a more complex scenario. Specifically, treatment of the SOD1G37R ALS mouse model with Cu^II^(atsm), a copper-targeting compound, resulted in improvements in survival and motor function in parallel with an elevation in mutant SOD1 protein levels [14]. Whilst seemingly paradoxical, the SOD1 was actually converted from metal-deficient to a more stable copper-replete form (holo-SOD1). These data supported the notion that the toxicity of SOD1 may be driven more by its metal content rather than by the level of the mutant protein itself. Together with other literature demonstrating that the pro-oxidant effect of zinc-deficient SOD1 can be modulated by the removal of copper [27], these data suggest that the metalation state of SOD1 is a key determinant of its function/toxicity.

In this body of work then, we have explored the metalloproteome across age in the ZnT3 KO mouse model, which may represent a phenocopy of AD or advanced ageing. There are a number of limitations of this work. Firstly, these data are snapshots in time and do not necessarily reflect the dynamic change in metals or the metalloproteome that may be occurring across age. One related consideration in this regard is the potential confound introduced by dietary metal content. The long-term modulation of metal intake via the food/water can impact peripheral and central levels of that metal, as well as have flow-on effects for the levels of other metal species (discussed later). Such alterations can then impact both “normal” and “disease-related” cellular chemistry (including the regulation of metal binding proteins and specific pathological proteins), biological pathways and downstream behavioural function (e.g., [28,29,30,31,32]). In this study, however, where all animals have received the same standardized metal-replete diet, then this is unlikely to impact our current findings. Secondly, due to the concentration of zinc and its transporters, together with the relative functional significance of zinc in this area, then we have focused only on the hippocampus. Thirdly, we have not performed formal protein identification, and so our interpretation of the data is limited to known elution profiles/standards. Future work would be focused on understanding the specific protein/s represented by the specific peaks we have identified, and would also examine other brain regions. Finally, once proteins have been identified, expression levels will need to be assessed to understand whether the differences observed in this study are driven by a change in metalation of a given protein/s and/or a change in levels of a specific metal binding protein.

Despite these limitations, the data presented provide insight into the metallobiology of the ZnT3 KO animal. As anticipated, there are significant deficits in zinc associated proteins, with all three peaks (P1–P3) identified at three months of age showing significant deficits in zinc load in the ZnT3 KO animals, as compared to the age-matched wildtypes. There were only small and non-significant trends in these same peaks at six months of age. At 18 months however, the P1 fraction, which demonstrated the highest zinc load and greatest difference to wildtype at three months, again showed a robust and statistically significant difference with wildtype animals. Such potential changes in the metalation state of proteins may, together with changes in protein expression, have significant impact on zinc-dependent pathways, such as those related to synaptic plasticity that we have previously published [7,8]. Our historical data suggested that there were significant deficits in hippocampal zinc levels at both 3 and 6 months of age in the ZnT3 KO mice [8], as well as significant and dynamic alterations in a number of different proteins (with some showing increases/decreases at 3 months that were then either exacerbated or reversed at 6 months; these proteins aren’t necessarily “zinc binding” proteins, and so may not contribute to the SEC-ICP-MS traces reported here). In the absence of peaks with elution times that may correspond to “classic” high abundance zinc binding proteins such as SOD and metallothionein [15,33], then identification of the possible protein/s in the P1-P3 fractions is not possible without further characterisation.

Whilst changes in the zinc metalloproteome were anticipated, what is perhaps more surprising is that there were changes in the iron, and to a lesser extent the copper, metalloproteome as well. In the iron proteome it is likely that ferritin (P1) is altered at both six and eighteen months of age; whereas for copper the change in fraction P1 (unidentified protein) was only observed at three months of age. What is interesting is that, in contrast to zinc, both the iron and copper levels associated with specific metalloproteins were elevated in the ZnT3 KO mice. As noted earlier, this may reflect an increase in iron and copper binding to specific proteins (such as ferritin and transferrin for iron; and ceruloplasmin, SOD and metallothionein for copper) and/or an increase in expression of those specific proteins.

An interaction between metals, such as zinc/ copper and zinc/ iron, has been widely reported in the past. In the case of copper for example, there are several reports in the clinical literature where zinc supplementation in coeliac disease, sickle cell anemia, acrodermatitis enteropathica and Wilson’s disease results (potentially via an effect on proteins such as metallothionein) in severe copper deficiency [34,35,36,37]. Similarly, a study in Caco-2 cells demonstrated that there is an inverse relationship between zinc and iron such that increased levels of extracellular zinc applied to the cells results in decreasing intracellular iron levels [38]. The converse of this finding, where high iron levels impact on zinc absorption, have also been reported in humans (reviewed in [39]). In our case, the facile explanation for the data presented is that the loss of ZnT3, which results in decreased brain zinc concentrations, has resulted in a compensatory upregulation in specific ZIP proteins that are responsible for the import of zinc into the cytosol and which are known to be rapidly regulated in response to cellular zinc levels (increased under zinc-deficient conditions and vice versa; it should be noted however, that not all ZIPs are responsive to zinc in this way—ZIP5, for example, is regulated in the opposite way [40,41]). In the context of the ZnT3 KO mice then, as the ZIP transporters may not be exclusive transporters of zinc (with some of them also handling iron, manganese, copper and cadmium for example) [40], then the ZnT3 KO mice may have a parallel increase in other metals (such as iron and copper). As the levels and localization of such metals are tightly controlled in a cell and tissue-specific way, then an upregulation of their associated regulatory proteins is also not unexpected. If there is a long-term change in iron in the ZnT3 KO mouse, this may add further complexity to the interpretation of the apparent age-dependent phenotype present in these animals (particularly given that iron is associated with aging and cellular senescence [42,43,44]) and this may all have long-term implications for zinc deficiency conditions. Further study is required to characterize the metalloproteome changes that occur in response to brain zinc deficiency, both from the perspective of zinc and its regulatory partners, but also more broadly for effects on other metals such as iron.

## References

[1] Adlard P.A., Bush A.I. (2006). Metals and Alzheimer’s disease. J. Alzheimers Dis..

[2] Bitanihirwe B.K., Cunningham M.G. (2009). Zinc: The brain’s dark horse. Synapse.

[3] Sensi S.L., Paoletti P., Bush A.I., Sekler I. (2009). Zinc in the physiology and pathology of the CNS. Nat. Rev. Neurosci..

[4] Huang L., Tepaamorndech S. (2013). The SLC30 family of zinc transporters—A review of current understanding of their biological and pathophysiological roles. Mol. Aspects Med..

[5] Jeong J., Eide D.J. (2013). The SLC39 family of zinc transporters. Mol. Aspects Med..

[6] Adlard P.A., Cherny R.A., Finkelstein D.I., Gautier E., Robb E., Cortes M., Volitakis I., Liu X., Smith J.P., Perez K. (2008). Rapid restoration of cognition in Alzheimer’s transgenic mice with 8-hydroxy quinoline analogs is associated with decreased interstitial Abeta. Neuron.

[7] Adlard P.A., Parncutt J., Lal V., James S., Hare D., Doble P., Finkelstein D.I., Bush A.I. (2015). Metal chaperones prevent zinc-mediated cognitive decline. Neurobiol. Dis..

[8] Adlard P.A., Parncutt J.M., Finkelstein D.I., Bush A.I. (2010). Cognitive loss in zinc transporter-3 knock-out mice: A phenocopy for the synaptic and memory deficits of Alzheimer’s disease?. J. Neurosci..

[9] Adlard P.A., Sedjahtera A., Gunawan L., Bray L., Hare D., Lear J., Doble P., Bush A.I., Finkelstein D.I., Cherny R.A. (2014). A novel approach to rapidly prevent age-related cognitive decline. Aging Cell.

[10] Cole T.B., Wenzel H.J., Kafer K.E., Schwartzkroin P.A., Palmiter R.D. (1999). Elimination of zinc from synaptic vesicles in the intact mouse brain by disruption of the ZnT3 gene. Proc. Natl. Acad. Sci. USA.

[11] Lee J.Y., Cole T.B., Palmiter R.D., Suh S.W., Koh J.Y. (2002). Contribution by synaptic zinc to the gender-disparate plaque formation in human Swedish mutant APP transgenic mice. Proc. Natl. Acad. Sci. USA.

[12] Linkous D.H., Flinn J.M., Koh J.Y., Lanzirotti A., Bertsch P.M., Jones B.F., Giblin L.J., Frederickson C.J. (2008). Evidence that the ZNT3 protein controls the total amount of elemental zinc in synaptic vesicles. J. Histochem. Cytochem..

[13] Palmiter R.D., Cole T.B., Quaife C.J., Findley S.D. (1996). ZnT-3, a putative transporter of zinc into synaptic vesicles. Proc. Natl. Acad. Sci. USA.

[14] Roberts B.R., Lim N.K., McAllum E.J., Donnelly P.S., Hare D.J., Doble P.A., Turner B.J., Price K.A., Lim S.C., Paterson B.M. (2014). Oral treatment with Cu(II)(atsm) increases mutant SOD1 in vivo but protects motor neurons and improves the phenotype of a transgenic mouse model of amyotrophic lateral sclerosis. J. Neurosci..

[15] Lothian A., Roberts B.R. (2016). Standards for Quantitative Metalloproteomic Analysis Using Size Exclusion ICP-MS. J. Vis. Exp..

[16] Royston J.P. (1982). Algorithm AS 181: The W-test for Normality. Appl. Stat..

[17] Thackray S.E., McAllister B.B., Dyck R.H. (2017). Behavioral characterization of female zinc transporter 3 (ZnT3) knockout mice. Behav. Brain Res..

[18] Portbury S.D., Adlard P.A. (2017). Zinc Signal in Brain Diseases. Int. J. Mol. Sci..

[19] Besser L., Chorin E., Sekler I., Silverman W.F., Atkin S., Russell J.T., Hershfinkel M. (2009). Synaptically released zinc triggers metabotropic signaling via a zinc-sensing receptor in the hippocampus. J. Neurosci..

[20] Huang Y.Z., Pan E., Xiong Z.Q., McNamara J.O. (2008). Zinc-mediated transactivation of TrkB potentiates the hippocampal mossy fiber-CA3 pyramid synapse. Neuron.

[21] Lee J.Y., Kim Y.J., Kim T.Y., Koh J.Y., Kim Y.H. (2008). Essential role for zinc-triggered p75NTR activation in preconditioning neuroprotection. J. Neurosci..

[22] Paoletti P., Vergnano A.M., Barbour B., Casado M. (2009). Zinc at glutamatergic synapses. Neuroscience.

[23] Meloni G., Sonois V., Delaine T., Guilloreau L., Gillet A., Teissie J., Faller P., Vasak M. (2008). Metal swap between Zn7-metallothionein-3 and amyloid-beta-Cu protects against amyloid-beta toxicity. Nat. Chem. Biol..

[24] Oskarsson B., Gendron T.F., Staff N.P. (2018). Amyotrophic Lateral Sclerosis: An Update for 2018. Mayo Clin. Proc..

[25] Mathis S., Goizet C., Soulages A., Vallat J.M., Masson G.L. (2019). Genetics of amyotrophic lateral sclerosis: A review. J. Neurol. Sci..

[26] Zucchi E., Ticozzi N., Mandrioli J. (2019). Psychiatric Symptoms in Amyotrophic Lateral Sclerosis: Beyond a Motor Neuron Disorder. Front. Neurosci..

[27] Estevez A.G., Crow J.P., Sampson J.B., Reiter C., Zhuang Y., Richardson G.J., Tarpey M.M., Barbeito L., Beckman J.S. (1999). Induction of nitric oxide-dependent apoptosis in motor neurons by zinc-deficient superoxide dismutase. Science.

[28] Adamo A.M., Liu X., Mathieu P., Nuttall J.R., Supasai S., Oteiza P.I. (2019). Early Developmental Marginal Zinc Deficiency Affects Neurogenesis Decreasing Neuronal Number and Altering Neuronal Specification in the Adult Rat Brain. Front. Cell Neurosci..

[29] Ayton S., Lei P., Appukuttan A.T., Renoir T., Foliaki S., Chen F., Adlard P.A., Hannan A.J., Bush A.I. (2019). Brain Zinc Deficiency Exacerbates Cognitive Decline in the R6/1 Model of Huntington’s Disease. Neurotherapeutics.

[30] Flinn J.M., Bozzelli P.L., Adlard P.A., Railey A.M. (2014). Spatial memory deficits in a mouse model of late-onset Alzheimer’s disease are caused by zinc supplementation and correlate with amyloid-beta levels. Front. Aging Neurosci..

[31] Flinn J.M., Hunter D., Linkous D.H., Lanzirotti A., Smith L.N., Brightwell J., Jones B.F. (2005). Enhanced zinc consumption causes memory deficits and increased brain levels of zinc. Physiol. Behav..

[32] Linkous D.H., Adlard P.A., Wanschura P.B., Conko K.M., Flinn J.M. (2009). The effects of enhanced zinc on spatial memory and plaque formation in transgenic mice. J. Alzheimers Dis..

[33] Kameo S., Nakai K., Naganuma A., Koyama P.B., Satoh H. (2014). Simple Analysis Method for Metallothionein-1, -2 and -3 in the Brain by One-Step Size-Exclusion Column HPLC On-Line Coupling with Inductively Coupled Plasma Mass Spectrometry. J. Anal. Bioanal. Tech..

[34] Cai S., Gong J.Y., Yang J., Wang J.S. (2019). Anemia following zinc treatment for Wilson’s disease: A case report and literature review. BMC Gastroenterol..

[35] Hoogenraad T.U., Dekker A.W., van den Hamer C.J. (1985). Copper responsive anemia, induced by oral zinc therapy in a patient with acrodermatitis enteropathica. Sci. Total. Environ..

[36] Porter K.G., McMaster D., Elmes M.E., Love A.H. (1977). Anaemia and low serum-copper during zinc therapy. Lancet.

[37] Prasad A.S., Brewer G.J., Schoomaker E.B., Rabbani P. (1978). Hypocupremia induced by zinc therapy in adults. JAMA.

[38] Arredondo M., Martinez R., Nunez M.T., Ruz M., Olivares M. (2006). Inhibition of iron and copper uptake by iron, copper and zinc. Biol. Res..

[39] Whittaker P. (1998). Iron and zinc interactions in humans. Am. J. Clin. Nutr..

[40] Kambe T., Tsuji T., Hashimoto A., Itsumura N. (2015). The Physiological, Biochemical, and Molecular Roles of Zinc Transporters in Zinc Homeostasis and Metabolism. Physiol. Rev..

[41] Weaver B.P., Andrews G.K. (2012). Regulation of zinc-responsive Slc39a5 (Zip5) translation is mediated by conserved elements in the 3’-untranslated region. Biometals.

[42] James S.A., Roberts B.R., Hare D.J., de Jonge M.D., Birchall I.E., Jenkins N.L., Cherny R.A., Bush A.I., McColl G. (2015). Direct in vivo imaging of ferrous iron dyshomeostasis in ageing Caenorhabditis elegans. Chem. Sci..

[43] Klang I.M., Schilling B., Sorensen D.J., Sahu A.K., Kapahi P., Andersen J.K., Swoboda P., Killilea D.W., Gibson B.W., Lithgow G.J. (2014). Iron promotes protein insolubility and aging in *C. elegans*. Aging (Albany NY).

[44] Masaldan S., Clatworthy S.A.S., Gamell C., Meggyesy P.M., Rigopoulos A.T., Haupt S., Haupt Y., Denoyer D., Adlard P.A., Bush A.I. (2018). Iron accumulation in senescent cells is coupled with impaired ferritinophagy and inhibition of ferroptosis. Redox Biol..

